# Neuro-Exergaming for College Students with Symptoms of Attention Deficit Hyperactivity Disorder (ADHD): Cognitive Benefits of an Acute Bout of Pedal-n-Play Interactive Physical and Cognitive Exercise

**DOI:** 10.3390/neurosci7040076

**Published:** 2026-07-01

**Authors:** Clara R. LaCorte, Mya C. Delesdernier, Cay Anderson-Hanley

**Affiliations:** Neuroscience Program, Department of Psychology, Union College, 807 Union Street, Schenectady, NY 12308, USA

**Keywords:** ADHD, exercise, executive functioning, working memory, attention, neuro-exergaming, neuropsychological assessment

## Abstract

This study investigated whether neuro-exergaming with an interactive physical and cognitive exercise system (iPACES), might alleviate symptoms in college students with symptoms of Attention Deficit Hyperactivity Disorder (sxADHD). It was hypothesized that challenges with attention and executive function, often experienced by those with sxADHD, would improve after an acute bout of pedal-n-play exercise. College students (*n* = 33; 18 with sxADHD) participated in a 20 min single bout of exercise, pedaling along a virtual pathway (tour), while steering the tablet. Mental exercise included a working memory (focus) task to steer toward assigned locations along the path. An exploratory experimental condition was also embedded in the basic pre/post design, wherein half of the students were randomly assigned collectibles (coins) in the pathway. Cognition was assessed (e.g., paper and digital Stroop, Trails, Digit Span) before and after the acute bout of exercise. Paired *t*-tests revealed significant improvements in executive function on both paper and electronic Stroop tasks for those with sxADHD, while significant change was also seen on Trails and Digit Span for normative students. Surprisingly, those with sxADHD, assigned to the experimental “multi-tasking” collectible (coin) challenge, performed significantly better on tests of executive functioning than normative peers, who improved more without coins. It is hypothesized that the collectible challenge provided additional mental stimulation or reward needed to increase attentional focus for those with sxADHD, leading to improved performance, post-exercise. While the findings of this study are preliminary, additional research can further explore possible designs for combined, interactive mental and physical exercise challenges, as well as further possible synergistic or differential neural activation, in order to maximize outcomes from the same amount of time exercising. Additionally, future research could examine longer-term use of neuro-exergaming, through a clinical trial, as an alternative to, or in conjunction with, medication for sxADHD treatment and symptom relief.

## 1. Introduction

### 1.1. ADHD Overview

Interventions have long been sought to address the challenges of Attention Deficit Hyperactivity Disorder (ADHD), a neurodevelopmental diagnosis that typically includes difficulties with attention and focusing, hyperactivity, and impulse control. It is estimated that over seven million children in the United States have been diagnosed with ADHD [1]. That number is just about 10% of children in the country. ADHD affects children across all socioeconomic backgrounds; however, it is diagnosed more frequently in boys (15%) than in girls (8%) [1]. Individuals with ADHD have impairments in their ability to sustain attention and organization [2]. Although ADHD is typically diagnosed during childhood, it is a lifelong condition that affects functioning long into adulthood. The current estimates of prevalence of ADHD in adults ranges from 2 to 6% [3]. ADHD affects the lives of children and adults, resulting in setbacks throughout numerous domains of life including socially, developmentally, academically, and in the workplace. According to the results of a 2017 study, individuals diagnosed with ADHD often struggle with tasks involving managing working memory, organization, planning ahead, sustaining attention, and controlling impulses [4]. Numerous studies have found that the academic performance of students with ADHD is often hindered from difficulty maintaining focus and organized thought processes. There has been no pin point to the exact cause of ADHD, but various potential factors contributing to this neurodevelopmental disorder have been examined by researchers, including certain genetic elements. Research findings have indicated that children with ADHD may have experienced the following factors: brain injuries, adverse environmental exposures during pregnancy or early infancy, maternal substance use during pregnancy, premature birth, and low birth weight but there is no concrete evidence supporting this [4]. While ADHD affects children across all socioeconomic classes, those with low socioeconomic status were 6.2 times more at risk for ADHD than children with higher socioeconomic status [5]. This is suggestive of some kind of external environmental influence and there is evidence to look further into the role that environment and parental involvement may have on ADHD in children. The current approach to treating ADHD typically combines medication and behavioral therapy. For children and adolescents above the age of 6, it is recommended by the American Academy of Pediatrics (AAP) that they receive behavioral therapy alongside medication for the most effective outcome [6]. According to a national survey sent out to parents in 2016, 77% (or 3 in every 4) children with ADHD were receiving treatment for their diagnosis. Of these children, 30% were treated with just medication, 15% were treated with some sort of behavioral intervention, and 32% were treated with both medication and behavioral therapy [6]. Yet medications can have unintended side effects and these strategies alone or even in combination, do not always yield the desired results [4]. Many children with ADHD, even after having treatment, receive special services including in school accommodations, peer interventions, and social skills training [6]. More needs to be done to help people struggling with ADHD find innovative and creative ways to alleviate their symptoms. Remaining on psychostimulant medications for long periods of time is not sustainable, they are expensive and thus not accessible to all, and furthermore can have serious health implications. Previous research has found that there is no improvement in cognition (attention and working memory) after prolonged usage of ADHD medication [7]. This has sparked an interest among researchers in the field to develop new first line approaches to treatment, most of them being non pharmacological, that are sustainable and have lasting benefits on cognition.

### 1.2. Cognitive Impairments in ADHD

Neuropsychological testing on both children and adults with ADHD has revealed impairments in function across all cognitive domains [8]. Deficits in memory performance and accuracy, inhibition skills, and detections of errors have been reported [9]. Although not as frequent, impairments in motor skills and motor control have also been seen alongside an ADHD diagnosis [10]. While there are widespread dysfunctions associated with ADHD, multiple studies have shown that two of the most prevalent cognitive impairments exhibited in ADHD are attention and memory [11]. Sub-categorial impairments of these domains include working memory (index and capacity), processing speed, attentional capacity, and selective and divided attention.

The cognitive domain of attention and concentration encompasses many different dimensions and is typically divided into two subdomains: selective attention and sustained attention. Concentration tends to align with sustained attention while divided attention is seen as a component of selective attention [12]. Selective attention is the ability to attend to and process relevant information while ignoring other non-relevant information that is being presented at the same time. Those who experience problems with attentional control tend to be extremely distracted and unable to shift their focus easily. Divided attention comes into play when an individual can successfully process both or prioritize the processing of information from two concurrent streams of information, usually an auditory stream and a visual stream. The inability to successfully complete this process is often present in common neuropsychiatric conditions. Sustained attention, or vigilance, is the ability for an individual to maintain focus and concentration while completing a certain task or activity over an extended period of time, even when distractions or other stimuli are present. The ability to sustain mental effort and resist the temptation of distractions is crucial to achieving vigilance. Sustained attention is an important factor for completing timely tasks, such as reading, studying, or doing a project [12].

Memory is an extremely complex and multi-faceted cognitive domain. For the purposes of this study, only working memory and processing speed will be addressed. Working memory is the capacity to hold information in consciousness for future use. Working memory is conceptualized into two different modalities, maintenance working memory and manipulation working memory. Maintenance working memory is the memory for verbal, spatial, and emotional information. The amount of information that can be processed is limited and the duration it can remain in maintenance working memory is limited as well [12]. Processing speed is the time it takes to perceive and respond to incoming stimuli and it is a reflection of how efficiently someone can process information [13].

### 1.3. Physical Exercise and ADHD

Physical activity has been shown to have numerous positive effects on the physical and mental health of children and adults. Alongside increasing quality of life and improving mood, there is strong evidence to suggest that regular physical activity results in enhanced cognitive brain function, subsequently having a positive effect on academic performance. A prominent hypothesis that supports this idea of an improvement in cognition is that there is an increase in blood and oxygen flow to the brain during exercise [13]. A 2022 study found that both acute and long-term physical activity were shown to have positive effects on relieving ADHD symptoms and supports the implementation of physical exercise as an adjunctive treatment intervention for ADHD [14]. Types of exercise that involve cardio activity, whether acute or chronic, have been shown to reduce impulsivity and improve response time in children with ADHD, with beneficial outcomes in attention being seen as well. Additionally, there are several other cognitive and behavioral benefits that come from both acute and chronic cardio exercise [15].

A 2011 study looked at the effects of single-bout interventions of aerobic exercise, video games, and exergaming on the performance of different cognitive control tasks. The results showed that single bout exercise on a treadmill may improve the ability to control cognition as a result of an increase in the ability to allocate attentional resources [16]. In a review of a study that analyzed the clinical benefits that physical activity has given individuals with ADHD, both the long-term and short-term effects of exercise on the cognition and symptoms of ADHD in children were studied. The results of this study found that in most instances, cognitive, behavioral, and physical symptoms of ADHD were somewhat reduced in individuals following an exercise intervention. The results also suggested that the use of exercise for ADHD is a useful intervention that does not appear to create any adverse effects [17]. Young adults with high scores on self-reported scales of ADHD saw improvements in their working memory performance immediately following varied acute exercise interventions [18]. The results of that study suggested that exercise can increase blood flow and the release of dopamine to the certain brain region responsible for working memory.

### 1.4. Mental Exercise and ADHD

Cognitive training or mental exercise is the engagement in regular activities that support varying cognitive abilities. Examples of cognitive training tasks include playing video games, doing puzzles, and learning new skills. Results of an existing study suggest an improvement in working memory performance after children with ADHD engaged in cognitive training [19]. Existing literature that examined the effectiveness of mindfulness-based interventions for treating ADHD beyond just the core symptoms, found that when used in conjunction with other interventions, mindfulness has the potential to be useful aids of symptom relief [20].

### 1.5. Exergaming

Exergames are potentially more engaging and motivating forms of exercise that may combine both physical and mental exercise. During exercise, off-the-shelf video games designed for entertainment may be synced up to exercise equipment, for example, a stationary bike [21]. Cognitively and physically demanding exergaming increased executive function performance in children with ADHD over an 8-week exergame intervention trial [22]. In our own lab, we previously investigated cognitive benefits of a single bout of exergaming for youth on the autism spectrum, which often overlaps with ADHD, exhibiting challenges with executive function [23], and we did find increased performance on executive function (Digit Span backwards) following a single bout of exergaming [24].

Neuro-exergames are exergames that are prospectively designed to address specific neuropsychological challenges. The iPACES (Interactive Physical and Cognitive Exercise System) was developed in our lab in response to encouraging preliminary research above. Since off-the-shelf exergames are designed primarily for entertainment and motivation, the mental challenges within are often ambiguous, cannot be adjusted, and are difficult to quantify. We sought to prospectively design and control the mental exercises, so as to embed specific and quantifiable interactive mental challenges, within the physical exercise experience. Thus, the iPACES is a pedal-n-play, exergame, that has been developed to primarily address executive function interactive with cardio physical activities, and initial research is encouraging for addressing executive function in older adults with Mild Cognitive Impairment (MCI) [25,26]. A key feature of the iPACES intervention is the emphasis on “interactivity” wherein pedal-n-play, indicates a fully integrated mental and physical exercise experience, such that pedaling controls on-screen progress, and steering the digital display device (e.g., tablet or smartphone) allows registration of mental exercise responses (e.g., choices among on-screen stimuli). The fully interactive approach of iPACES is intentionally synergistic, facilitating mind and body to work in sync. This interactive approach is in contrast to some protocols which utilize a simultaneous-only or “dual-task” approach wherein mentation and physical activity are not in sync, but rather at odds with one another (e.g., walking while doing a non-interactive, disparate task such as counting backwards, which has been shown to compromise both mental and physical performance, rather than facilitating improvement). The interactivity of iPACES is hypothesized to facilitate optimal brain health by utilizing the synergy of mental and physical challenges, via naturalistic-styled tasks that may activate neural networks similar to real-world path-navigation scenarios (e.g., hunter-gatherer, seek-find activities).

Previous studies involving iPACES as a neuro-exergaming intervention have shown promising effects for executive function, in both single bout and long-term interventions, primarily with older adults. Using iPACES led to significant benefits to executive functioning of older adults dealing with mild cognitive impairment [25,26]. Older adults with MCI experienced significantly greater improvements in executive function compared to a normative sample of older adults [27]. This research aims to extend the investigation of potential benefits of neuro-exergaming to youth with symptoms of ADHD (sxADHD), to see if similar neural activation through interactive mental and physical exercise, can yield similar benefits to executive function. Given prior pilot work indicating the feasibility of using iPACES pedal-n-play, with youth with various neurodevelopmental challenges, including ADHD [28], we sought to conduct a single bout study with young adults both with and without ADHD, and to examine the impact of some prescribed variation in on-screen mental challenges.

### 1.6. This Experiment

The goal of this single-bout study was to attempt to replicate the promising existing literature on exergaming for addressing executive function challenges in varied populations, and specifically to extend the encouraging research using pedal-n-play neuro-exergaming to those with sxADHD. Considering the known challenges that students with ADHD face in certain cognitive domains, particularly attention and executive function, results of this study may be useful for curate behavioral intervention plans to alleviate and/or treat certain symptoms of ADHD among adolescents. These findings might also be used to generate further and more comprehensive research on using this type of intervention as an alternative to medication or alongside medication as treatment for ADHD. Specifically, implementing frequent interactive gaming that involves a combination of physical and mental exercise for students struggling with sxADHD as a way to improve their focus and enhance their ability to succeed in academic environments.

### 1.7. Hypotheses

Following a single bout of neuro-exergaming (20 min) with iPACES:

- Primary hypothesis: executive function will be significantly improved for those with sxADHD.
  - this effect should exceed known practice effects;
  - this effect may exceed any benefit to normative participants;
  - those randomly assigned to an additional, embedded “multi-tasking” mental challenge (coin collectibles) may reduce benefit to executive function.
- Exploratory analyses: measures of attention will also be examined.

## 2. Materials and Methods

### 2.1. Participants

The sample (*n* = 33) consisted of undergraduate students at a liberal arts college in the northeastern United States. Seven participants disclosed having a diagnosis of ADHD, eleven additional participants scored consistent with ADHD on the Adult ADHD Self-Report Scale (ASRS-vI.I), and 16 participants had no diagnosis or indication of ADHD symptoms. Participants were volunteers who were recruited to the study through public posting of the study. The risks and benefits of the study were reviewed with each participant who signed an informed consent form approved by the college’s Institutional Review Board (IRB). The study took place in a college laboratory for 1.5 h; participants had the option to receive either a cash payment ($24 similar to current hourly minimum wage rate) or receive out of class activity credits required for a psychology course. Demographics can be viewed in Table 1.

### 2.2. Procedures

Participants came to the Healthy Aging & Neuropsychology Research Lab for 1.5 h to complete the protocol, including a variety of tests of cognition detailed below (e.g., paper and electronic Stroop, Trail Making, Digit Span), completed before and after a 20 min single bout of pedal-n-play exercise using iPACES app (v3) on an android tablet, paired with an under-desk pedaler. The primary “focus” mode of the iPACES screen experience (on an android tablet), included pedaling along a virtual bike-path, in this case, a canal-way through an amusement park, while steering toward assigned locations along the way (e.g., rollercoaster), and then reversing direction to steer toward each of same locations, but in reverse order. An additional “collectible” challenge was randomly toggled on for half of the participants, wherein coins could be “tagged” by steering through them along the pathway between locations. This additional feature had been programmed in for entertainment purposes, and it was assumed that while some participants would like the added “eye candy” replete with audio reward (“cha-ching”), it was assumed that collectibles would be distracting from the core memory-reversal task and could reduce performance through interference. Participants were randomly assigned to one of these two variations in the intervention (coins: on or off).

#### The iPACES System (v3.0)

**Figure 1:**
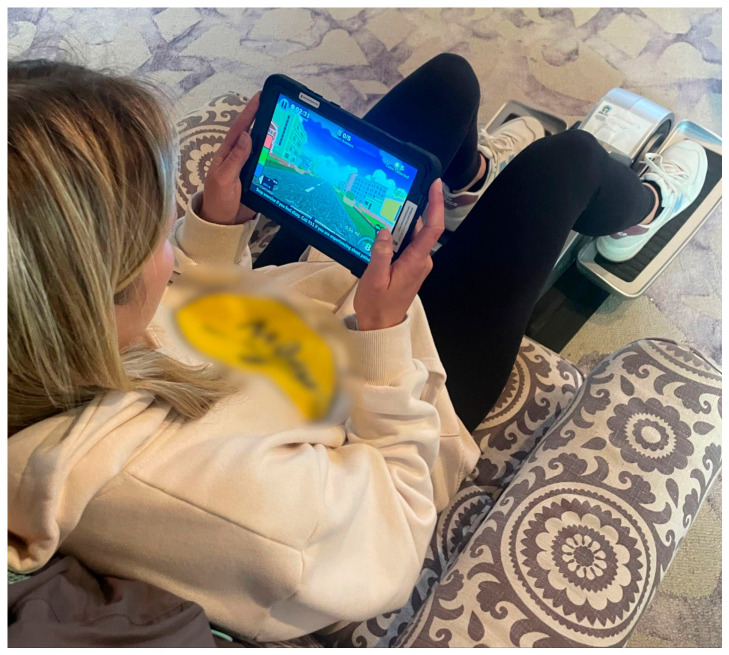
Pedal-n-play neuro-exergaming with the interactive physical and cognitive exercise system (iPACES).

The components of the interactive physical and cognitive exercise system (iPACES) include an android tablet with version 3 of the iPACES application installed, a heart rate (HR) monitor (e.g., CooSpo HR band) with Bluetooth connection to the app on the tablet, and a Cubii under-desk elliptical, which is used as a stationary pedaler (see Figure 1). All participants pedaled at a resistance level of “2.” Heart rate data was collected at 5 min intervals throughout the 20 min intervention. Once the user was situated and in a comfortable position, a 1 min warm-up training session allowed participants to get familiar with the virtual scenery and steering by steering to tag coins in the pathway. After the warm-up, participants were presented a list of “locations” to visit along the virtual path; and were asked to read these aloud to ensure comprehension and facilitate recall. The participant then begins pedaling along a path. How fast they move down the path is controlled by how fast the participant is pedaling.

**Figure 2:**
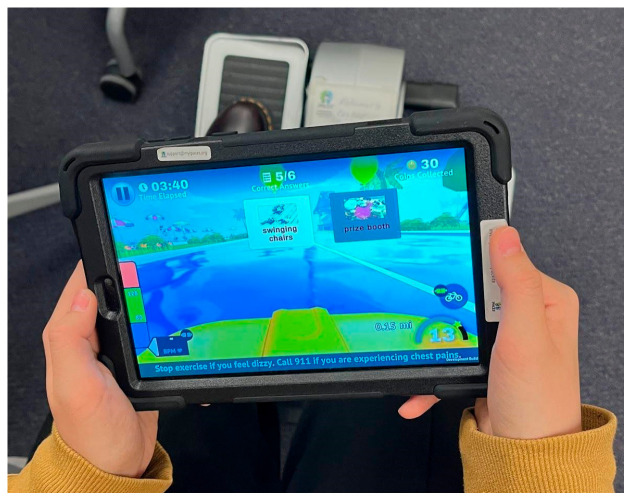
Example iPACES virtual bike path, herein featuring a canal-way through an amusement park.

After several seconds have passed, the user is presented with two words along their path, one on the left side of the screen and one on the right side. The goal of the game is to steer towards the word that was part of the list the participant was asked to remember moments before. The steering of the game is controlled by the participant tilting the tablet left to go towards the word on the left side and right to go towards the word on the right side. The task is completed correctly when the participant steers in the direction of the correct word. During the warm up round, coins randomly appeared on the path and the participants were to steer towards them to collect them in between the words. For the actual intervention, participants were randomly assigned to have coins “on” to be collected during the game, or “off” to not have any appear. The differentiation between coins on and off was not analyzed. After the 60 s is complete, the participant begins the real trial. The iPACES app has a variety of different “themes” for the participant to pedal in, and for this intervention, the waterpark version was chosen. The initial list of words contains three items. The participant is to successfully move forward in the game by correctly identifying, in the same order as presented, all locations to visit (presented as typed words with accompanying photo, such as “rollercoaster”). The participant then is challenged to correctly identify the same list of locations again, this time in “reverse order” from the original presentation. Once this has been done without error for two different lists of three words, the list is increased to four items for another two trials, before progressing to five items, and so on. An example of a participant engaging in the iPACES game is shown in Figure 2.

The pre-intervention assessment of neuropsychological functions began with a digital version of the Stroop task (electronic Stroop or eStroop), integrated into the iPACES app and completed on the study tablet. Next, participants completed a paper Stroop task, followed by the Color Trails Test and finally the Digit Span Test. Participants then began the 20 min single-bout exergaming intervention where they interactively played with iPACES on a tablet and pedaled an under-desk-style elliptical pedaler. After the intervention was complete, the participants began the post-intervention assessment which again included alternate forms of the eStroop task, Paper Stroop Task, Color Trails Test, and Digit Span Test, respectively. Results were either recorded by the researcher on a participant response sheet or digitally captured in the iPACES server system using an ID.

After completion of the intervention and cognitive measures, participants filled out a Google Form which included the Exercise-Induced Feelings Inventory (EFI), Flow State Scale, the Adult ADHD Self-Report Scale (ASRS-vI.I), and a demographics questionnaire.

### 2.3. Measures

As noted above, cognitive measures were administered before and after the acute bout of exercise. Where available participants were randomly assigned to alternate forms of the neuropsychological tests to reduce practice effects with repeated pre/post assessments.

#### 2.3.1. Paper Stroop Task, 40-Item Version

The Paper Stroop Task is a common neuropsychological test that includes three trials, a color block trial, a word reading trial, and a color word interference trial. The Paper Stroop Color trial is a measure of selective attention and working memory, while the Paper Stroop Word trial is a measure of visual processing speed [29]. A shortened version of the traditional task was used in this study which included 40 items per trial and has good reliability and validity [30]. For each trial, the participants were presented with a piece of paper that had all 40 stimuli listed, ten items across and four lines down. Above the trial was a practice sample that included ten items so the participant could be familiar with the task before completing it. In trial A, color blocks, the stimuli are colored blocks that are presented to the participant in either the color red, blue, or green. The participants are to say the color of all 40 blocks as quickly and accurately as they can. In trial B, the word reading trial, the participant is presented with 40 written words, either “red,” “blue,” or “green,” and expected to read the words out loud as quickly and accurately as possible. Data from the Paper Stroop Tasks were recorded in seconds and is reported as a timed score.

#### 2.3.2. Electronic Stroop Task (eStroop; iPACES LLC, Clifton Park, NY, USA)

The eStroop task is a component of the iPACES (v3) application, with good initial reliability and validity [31], and offers four different tasks to assess neuropsychological functions. This task differs from the traditional paper Stroop task in a few ways. First, no instructions are read aloud to the participant. They are to read the prompts on the screen and comprehend the task for themselves. Second, there are two grids of stimuli presentation for each of the four trials. The stimuli are also presented one at a time instead of all at once; therefore, a response is needed from the previous stimuli to reveal the subsequent one. Additionally, participants indicate a response to the stimulus by tapping the “red” or “blue” button on the bottom of the screen, compared to saying the response out loud in the paper Stroop tasks. One difference between the electronic and paper versions of the Stroop task is that the electronic version only uses two different options for color stimuli, red and blue, while the paper version uses three. Consistent with traditional paper’s Stroop tasks, there are the standard, color, word, and color-word/interference trials, but there is also an additional, 4th trial (a second experimental, interference trial) wherein the color of “ink” (screen font) used to display the words, sometimes matches the word being presented, rather than always being incongruent as in the 3rd trial. Once the task has begun, the stimuli are presented on the screen, 20 items at a time, arranged in a four by five grid configuration. Each trial has a total of 40 stimuli, but they are presented in two blocks. Data from the eStroop task is reported as average stimulus reaction time, in milliseconds; which is then converted to total time in seconds.

#### 2.3.3. Color Trails Test (PAR, Inc., Lutz, FL, USA)

The Color Trails Test is a two-part task that was adapted from the classic black and white Trail Making Test; instead, using colors and numbers rather than letters and numbers (to increase cross-cultural accessibility). Color Trails Test 1 is a neuropsychological measure of divided and sustained attention with good reliability and validity [32]. Color Trails 1 is administered first, and participants are given a sheet of paper containing 25 circles, even numbers are in yellow circles and odd numbers are in pink circles. Before completing the timed trial, there is a smaller scaled practice version on the other side of the paper with eight numbers. The goal is to draw a line connecting the numbers in numerical order, without lifting the pencil off the paper, as quickly and accurately as possible.

#### 2.3.4. Digit Span Test (Wechsler Adult Intelligence Scale, 4th ed.; Pearson Assessments; Broomfield, CO, USA)

The Digit Span Test is a common measure of an individual’s immediate verbal recall span. There are two components to the test, Digits Forward and Digits Backward. They can be totaled together to come up with a Digit Span Total Score. In particular, the Digit Span Forward Test is a measure of verbal working memory and short-term memory. This test is unique because it is a measure of the efficiency of attention, rather than strictly being a measure of memory. The Digit Span Total Score is representative of overall broader cognitive abilities and attentional capacity, and has good reliability and validity [32]. For Digits Forward, participants are read a set of numbers and asked to repeat it back exactly as they heard it. The set of numbers starts off with two items and increases up to nine items. Every correct answer yields one point, for a total of 16 points. For Digits Backward, participants are read a set of numbers and then asked to repeat them back in reverse order. The set of numbers starts off with two items and increases up to eight items. Every correct answer yields one point, for a total of 14 points. The Digit Total Score is calculated by adding Digits Forward score to the Digits Backward score, and it is out of 30 points.

#### 2.3.5. The Exercise-Induced Feelings Inventory (EIFI)

The Exercise-Induced Feeling Inventory is a 12-item measurement of feelings that occur while someone is engaging in physical activity. The measure has good reliability and validity, and identifies four specific states of feeling: physical exhaustion, positive engagement, revitalization, and tranquility [33]. Participants are presented with 12 adjectives and asked to rank how much they experienced that specific feeling during their recent exercise session. The scale contains five items: 0 to 4, Do Not Feel to Feel Very Strongly, respectively. Scores are summed for each category based on the responses to the questions associated with it.

#### 2.3.6. The Flow State Scale (16-Item Version; Mind Garden, Inc.; Menlo Park, CA)

The Flow State Scale is a 16-item measurement of flow that has good reliability and validity [34]. Participants are presented with 16 statements and asked to rank them on a 5-point scale that ranges from 1 (strongly disagree) to 5 (strongly agree). A total flow score is calculated by summing the responses of all 16 questions together.

#### 2.3.7. Adult ADHD Self-Report Scale (ASRS-vI.I)

The Adult ADHD Self-Report Scale (ASRS-vI.I) is an 18-item questionnaire with good reliability and validity [35] that asks participants to respond to statements based on how they have felt over the past six months. Each statement has five possible responses: never, rarely, sometimes, often, and very often. The 18 statements were developed to reflect the diagnosis criteria laid out in the DSM-5. There are two parts to the scale, Part A which includes the first six questions, and Part B which includes the remaining twelve questions. Part A includes the most predictive questions and a score of four or more (out of six) indicates high consistency with ADHD [35]. Participants were given an electronic copy of this questionnaire to fill out via Google Forms.

#### 2.3.8. Statistical Analysis

The collected data was organized in Google Sheets and analyzed using the Statistical Package for the Social Sciences (SPSS v. 12.0). The main hypotheses were tested via paired *t*-test, and repeated measures ANOVA to evaluate the interaction between time (pre/post) and group (ADHD/normative). Additional, exploratory analyses were conducted via *t*-tests for pre/post comparisons.

## 3. Results

Thirty-three undergraduate students completed a 20 min single bout of pedal-n-play neuro-exergaming with iPACES. The average age was 19.6 years (SD = 1.2; range = 18–22), and average years of completed education for the sample was 13.2 (SD = 1.2; range = 12–15). The data set was categorized, based on the presence of diagnosed and/or self-rated ADHD. Some participants reported having a diagnosis of ADHD (*n* = 7), and/or others scored on the self-report scale highly consistent with ADHD (>4; *n* = 11). The combined sample is collectively referred to as having symptoms of ADHD (sxADHD) (*n* = 18) and was the primary comparison group for main analyses, while the smaller ADHD (as diagnosed) subgroup scores are also presented as well. Participants classified as “normative” (*n* = 15) had not received a diagnosis and did not self-score as having ADHD. It is noteworthy that there were no significant differences between the diagnosed vs. self-reported ADHD students on demographic or baseline variables; however, given that there were only women in the diagnosed ADHD group, we also re-ran the comparison dropping the five men in the self-reported ADHD group, to double-check in case they had any significant differential sway on the group, but there were still no significant differences. Additionally, randomization to coins on/off conditions appeared to work well in that a roughly equal split of the diagnosed ADHD individuals was assigned to each condition (4 and 3). Similarly, it was reassuring that there were also no significant differences in the demographic or baseline variable between the coins on/off conditions. Average heart rate during the single bout of exercise was similar across the three groups (no significant difference). Additional remaining demographic statistics can be seen in Table 1.

Data from main outcome variables were screened for assumptions. Some deviation from normality was observed, especially in the Stroop tasks with some skewing, which is consistent with what is found in the research literature. While repeated measures ANOVA can still be robust, even with moderate deviations from normality, we reviewed the data for outliers, as well as surveying the Q-Q plots. Data were found to be within bounds and to conform adequately to the reference line.

**Figure 3:**
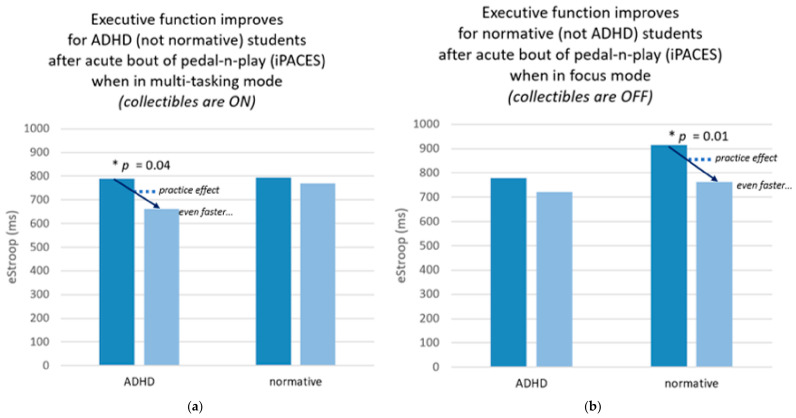
Figures showing the 3-way interaction found in exploratory analyses, revealing a differential effect on executive function for sxADHD vs. normative students, depending on whether coin collection was on or off: (**a**) sxADHD students improve more with coins on; (**b**) normative students improve more with coins off. Practice effect differential is shown and based on known effect size from merely repeating the test a second time without any intervention, thus illustrating here the effect post exercise appears to be “real” rather than simply an effect of familiarization with the test. Arrows indicate the direction of change is in the “beneficial” direction, with time to complete decreasing, thus comparatively “even faster”. * *p* < 0.05.

Primary hypotheses:

(1) sxADHD within subjects: From pre- to post-exercise bout, those with ADHD improved significantly on two of the four tests of executive function (see Table 1: pStroopC and eStroopC; *p* = 0.002, and 0.03, respectively, while ColorTrails2 and DigitSpanBackwards were n.s.). Interestingly, while not an a priori hypothesis, it can also be seen in Table 1, that normative students improved significantly across all four measures.
(2) Normative comparison: A multivariate Repeated Measures Analysis of Variance (ANOVA) was conducted to examine possible differences between groups (sxADHD vs. normative) for the primary executive function measures (e.g., pStroopC, eStroopC, ColorTrails2, and DigitSpan Backwards). While we had hypothesized that any effect might be greater for those with sxADHD than normative students, we did not have strong theoretical backing or expectations, other than perhaps that the sxADHD students might have more room to improve and could therefore exhibit a greater effect. However, the preliminary within group pre–post-comparisons in Table 1 seemed to indicate a more significant and across the board improvement for normative than sxADHD students. While prior research with iPACES in our lab led to a moderate effect (*d* = 0.68; suggesting a sample of *n* = 68), this was for a different population (older adults) and herein was a preliminary chance to see whether any change for those with sxADHD was greater than that for normative participants. Given limitations in grant funding, the sample size was smaller than recommended per the power analysis; thus, it was not surprising that the overall multivariate interaction (time x sxADHD) was not significant [F(4, 27) = 0.30, *p* = 0.83)], and univariate interactions were also non-significant. However, this lack of interaction indicated the group differences were not exceptional and so the groups were collapsed in order to test for a possible main effect of time (in addition to the separate within group analyses in Table 1). Pair-wise (*t*-test) comparisons (pre/post) across all participants revealed significant main effects for time, with significant improvements found for: pStroopC (*p* < 0.001), eStroopC (*p* = 0.004), ColorTrails2 (*p* = 0.03), but not DigitSpanBackward (*p* = 0.64).
(3) Coins on/off: Exploratory hypothesis: a 3-way interaction (time × sxADHD × coinsON) was examined using multivariate Repeated Measures ANOVA and was found significant for sxADHD when the “multi-tasking” collectible challenge was turned on [F(4, 25) = 3.51; *p* = 0.02], while normative individuals had a significant effect when in “focus mode” without the collectibles [(F(4, 25) = 4.77; *p* = 0.005)]. This differential effect was seen most prominently for eStroopC (see Figure 3).
(4) Attention measures: While the effect of neuro-exergaming on tests of executive function was the primary focus of this study, attention measures are necessarily administered as part of the standardized procedures which include step-wise preparatory induction tasks for Stroop, Trails and Digit Span (e.g., Stroop A & B, Trails1, Digits Forward). Thus, we planned to examine these measures as well. Table 1 interestingly reveals significant changes pre to post for normative students across almost all precursor attention measures, while those with sxADHD only show change on StroopA subtests (colored blocks); this was significant for both the paper and electronic versions (*p* < 0.01 and <0.001, respectively).

## 4. Discussion

The primary objective of this single-bout pedal-n-play study was to extend prior research to a sample of participants with symptoms of ADHD (diagnosed and/or self-reported), given that prior research applying exergaming had yielded promising benefits to executive function for various neurological disorders, including Autism Spectrum Disorder (ASD [24]) and Mild Cognitive Impairment (MCI [25,26,27]). College students with sxADHD (*n* = 18) and normative students (*n* = 15) pedaled and steered along a virtual bike path completing a naturalistic mental challenge, for a 20 min acute bout with the neuro-exergame, iPACES (the interactive physical and cognitive exercise system). It was hypothesized that executive function would benefit, similar to prior studies. While no significant difference was found between the sxADHD and normative students, in terms of the effect of the bout on executive function (EF), instead EF was observed to improve significantly pre- to post-exercise on three of the four key measures (e.g., pStroopC, eStroopC, and Color Trails 2). Thus, this bump in EF was observed across the combined sample, and exceeded practice effects for these measures (see Table 1).

In an exploratory analysis of the possible impact of an additional mental task, multi-tasking to collect coins, amid the primary mental challenge of the iPACES pedal-n-play virtual tour, it was surprisingly found to enhance the effect for those with sxADHD, while normative individuals performed better without the apparent distraction of the collectible challenge. More research will be needed to replicate and tease apart the seemingly paradoxical benefit of the added coin challenge for those with sxADHD. Instead of distraction with a cost to cognitive functions, it appears that the multi-tasking feature provided enhanced engagement.

These findings are similar to the conclusions of another study which found that children with ASD significantly improved on an executive function task (Digit Span) upon completion of an exergaming intervention [24]; however, it is unclear why that specific measure did not significantly increase in this study. The prior finding may have been due in part to a younger age range wherein greater responsiveness on the Digit Span task might be expected. Although the two conditions, ADHD and ASD, are distinct, there are many overlapping components shared among them. These results align with previous literature that found enhancements to working memory performance in children with ADHD after they engaged with cognitive training [19] or exergaming [22]. Results from a recent study on an acute bout of exercise found improvements in executive functioning and perception after a 30 min cognitive and physical exercise intervention on children with neurodevelopmental disabilities [36].

Engagement in physical exercise, whether acute or chronic, has been shown to enhance cognitive brain functions and relieve symptoms of ADHD. A compelling hypothesis that supports this theory looks at the direct relationship between blood and oxygen flow to the brain and enhanced functioning [35]. An increase in blood flow to the brain could be a possible explanation as to why there were significant changes in performance for the ADHD participants. Additionally, there may be a synergy in the interactive mental and physical exercise that leads to enhanced neural activation, leading to improved outcomes. Further research will be needed to examine possible biomarker and neural correlates to corroborate these theories.

### 4.1. Strengths

The present study was designed in such a way that there are a number of strengths which helped to eliminate confounds and strengthen the internal validity of the experiment. The first thing taken into consideration was the mere exposure effect and practice effects that would come from repeated presentation of the same neuropsychological tests in a short period of time. In an effort to reduce both of these effects, alternate forms of the same assessments were used for the pre-intervention assessment and post-intervention assessment.

The specific neuropsychological tests were selected with careful detail to ensure the most accurate measurements. The Color Trails Test is an adaptation of the Trail Making Test, but uses alternating colors (pink and yellow), instead of letters of the English alphabet. That change makes the Color Trails Test a more inclusive and cross-cultural measure of cognitive function, and provides equal opportunity for success to participants who may not be as familiar with the English language as others. In addition to the paper Stroop Task, an electronic (digital) version was included in the neuropsychological assessment. The purpose of having two versions that assess the same cognitive function was to have greater validity, as well as account for the fact that many tasks in current practice are done online or in some digital format.

ADHD was measured two different ways in this study. The first method was a self-report of a diagnosis by a medical professional. The second way ADHD was measured was through a scored scale, enabling inclusion of persons who might be symptomatic, but have not pursued diagnosis.

### 4.2. Limitations

The above strength is also a limitation, in that there was a relatively small number of professionally diagnosed students with ADHD; it would be desirable for future research to recruit and evaluate a larger sample of those meeting strict DSM-5 criteria, perhaps confirming these preliminary findings. Another limitation in this study was that all participants did not pedal-n-play an identical version of iPACES, since roughly half of the participants completed the iPACES intervention with an additional collectible challenge of tagging coins turned on, meaning that as they were pedaling along the virtual path, coins would appear on the screen and they were instructed to steer towards the coins to collect them as part of the experience. Furthermore, there was some complexity in the random assignment of this sub-condition leading to some imbalance in the number of participants experiencing the collectible challenge, likely due to the fact that randomization was done before sxADHD categorization was known. While it was expected that this multi-tasking add-on would be distracting and limit benefits of the neuro-exergaming intervention, it appears that it may have been fortuitous since sxADHD participants seem to have benefited. Further research will be needed to try to replicate and understand this phenomenon.

Another potential limitation is the lack of characterization of possible subtypes of ADHD, such as with or without prominent hyperactivity. It is interesting to note that the baseline ADHD response times are sometimes much faster than normative participants, particularly for the digital eStroop tasks, than the paper version, perhaps indicating a potential confound in terms of engagement with type of materials that may be more stimulating in line with neural preferences. Similarly, the potential impact of prescribed medications is unclear. Future research might carefully query time of last dose, and include ratings of how well controlled one’s symptoms seem during the session.

Although partially accounted for with alternate forms, there is still a possibility that practice effects (pre to post) created familiarity with the neuropsychological tests for the participants, leading to significant improvements; however, it is reassuring that change scores observed herein exceed estimates of practice effects from other studies.

There were multiple research assistants who administered the protocol to participants during the period of data collection. Specific instructions and proper training was given to all lab assistants, but there is still a possibility of the protocol being carried out in slightly different ways. This can include the speed at which things were read to the participant, the tone of voice the researcher used, as well as their mood and affect. It is possible that there could have also been inconsistencies in how tasks were timed. Although the same stopwatch was used throughout data collection, it is possible that not every research assistant started and stopped it in the same manner.

Additional limitations for this experiment came from specific context of the college from which the data was collected. There was a maximum amount of funding that students were allowed to receive, and a minimum hourly payment that participants must be compensated for by participating in the study. This limited the amount of participants that could participate in the study. To balance this out, participants were able to complete this study for psychology course credit. Regardless of how participants were compensated, it is hard to know the internal motivation people have and if they were giving their best effort, or just trying to be finished as quickly as possible to get the reward. Time limitations were also a factor in the academic environment, in terms of not being able to conduct a long-term study with repeated intervention with follow-up vs. only a single-bout.

### 4.3. Future Research

The future direction of ADHD treatment remains far from fully developed. Substantial progress is still needed, with ongoing development required in areas such as psychotherapy, behavioral interventions, and improved access to care [36,37,38].

In terms of future research, a lot can be done to expand upon this preliminary pilot study to gather additional evidence that would lead to more generalizable results. The study could be recreated to provide a longer-term intervention with follow-up, or even the possibility of a longitudinal study to see if the results would replicate or if new results would emerge. The updated protocol could include more extensive measures of cognitive domains and include other types of assessments such as academic or extracurricular ones (sports, musical instruments, etc.) to see how these aspects of function are impacted. It could also control for medication use and evaluate how the impact seen here on executive function compares with medication alone.

Further inquiry for eStroop scores might be warranted for looking at the number of correct responses in addition to the response time. It is possible that people with ADHD perform these tasks faster because they are more impulsive, and it does not necessarily have anything to do with being more accurate and increasing performance.

There is potential for future research to analyze these results and curate them to be used in the future as treatment for ADHD either alongside medication and behavioral therapy or in lieu of it. Prescription medications are not sustainable for long-term use. While they can provide significant benefits in the interim, there are many negative side effects, addictive qualities, high prices, and people begin to become dependent on them. Having a set of behavioral interventions or tasks that improve attention and memory functions in people diagnosed with ADHD, possibly looking specifically at impulsiveness, would be extremely beneficial to the field of clinical neuroscience, especially within pediatrics.

## 5. Conclusions

Neuro-exergaming, that combines interactive physical and cognitive exercise, with dynamic virtual scenery and mental challenges, such as herein with pedal-n-play iPACES, shows promise in this single bout study, for addressing the executive function challenges of sxADHD. The design of these interventions needs more research as the effects of various gaming and physical challenges therein appear nuanced, as some entertainment or reward factors may be enhancing to some with sxADHD, while distracting to normative players. These preliminary findings are in line with a burgeoning and progressive research literature examining the cognitive benefits of varied forms of exercise for ADHD, including digital therapeutics such as exergaming, many synthesizing reports, as well as a refined mechanism of action research [39,40,41,42,43,44,45,46,47,48].

Along with this uptick in related research, there has also been increased analysis of the more nuanced factors of the types examined herein, that might contribute to most effective exercise prescriptions for ADHD [38,49,50,51,52,53,54]. Additionally, more research is needed on the role of gaming features, such as the coin collection challenge, and exploration of theoretical links to explain differential outcomes for those with ADHD who may be drawn to engage due to activation of a neural network reward system or potentially other links. For example, the Experiential Learning Theory, has been invoked to explain some connections between gaming and ADHD [55], while functional connectivity analysis has been used to map network-level brain dynamics during gameplay, revealing differential patterns depending on game type and gamification features [56]. There is much more to investigate and much more to be learned that may improve care of patients with ADHD. This is an exciting and hopeful period for improved non-pharmacological interventions, such as physical activity, including digital therapeutics and neuro-exergaming, specifically, to enhance the care and outcomes for those with ADHD.

## Figures and Tables

**Table 1:** Neuropsychological variables: Means, standard deviations, and significance of change from pre to post (single bout) by ADHD.

		Pre-Exercise Cognitive Performance						Post-Single Bout Exercise Cognitive Performance										Change (Post-Pre)		
		ADHD (Diagnosed)		sxADHD (dx + Survey)		Normative Students		ADHD (Diagnosed)			sxADHD (dx + Survey)			Normative Students			All	sxADHD	Normative	Practice Effect
		*n* = 7		*n* = 18 (7 + 11)		*n* = 15		*n* = 7			*n* = 18 (7 + 11)			*n* = 15			*n* = 33	*n* = 18	*n* = 15	
Demographics & Neuropsychological Tests	Cognitive Domain	Ave	SD	Ave	SD	Ave	SD	Ave	SD	*p* (*t*-Test)	Ave	SD	*p* (*t*-Test)	Ave	*SD*	*p* (*t*-Test)	*p* (*t*-Test)	Post-Pre	Post-Pre	Post-Pre
Age		19.9	0.69	19.7	1.32	19.3	1.11													
Sex (biological/at birth/male)		100%		72%		73%														
Gender (% nonconforming)		0%		0%		6%														
Race (% non-Caucasian/White incl. Asian, Black, other)		43%	0.53	33%	0.49	33%	0.49													
Hispanic		*n* = 1		*n* = 0		*n* = 1														
Education (years)		13.1	0.90	13.3	1.14	13.1	1.36													
Heart Rate (ave during acute bout)								103.5	8.9	0.07	97.1	10.8	0.51	94.1	15.2	0.15				
paper Stroop (pStroop) A (colored blocks)	attention	20.5	3.3	21.0	3.2	19.5	4.2	18.8	2.3	0.08	18.6	2.7	0.00	17.4	3.1	0.47	<0.001			
paper Stroop (pStroop) B (black words)	attention	17.5	4.4	17.1	3.2	15.2	2.6	16.4	1.9	0.32	16.2	2.0	0.12	14.6	2.6	<0.001	0.04			
paper Stroop (pStroop) C (incongruent colored words)	executive function	32.2	8.2	32.5	6.0	33.8	8.5	30.0	4.7	0.49	28.0	4.6	0.002	28.9	5.9	<0.001	<0.001	−4.5	−4.9	−3.7
pStroop A/C ratio	executive function	0.69	0.25	0.67	0.16	0.58	0.07	0.63	0.07	0.62	0.67	0.08	0.90	0.61	0.10	<0.001	0.53			
electronic Stroop (eStroop) A (colored blocks)	attention	26.2	5.2	25.5	3.4	25.1	3.9	23.4	4.5	0.01	22.7	3.1	<0.001	24.4	5.6	<0.001	0.01			
electronic Stroop (eStroop) B (black words)	attention	28.0	7.3	25.9	4.7	24.3	2.6	24.1	2.9	0.11	24.3	1.9	0.09	24.7	3.4	0.94	0.30			
electronic Stroop (eStroop) C (incongruent colored words)	executive function	31.8	7.3	31.4	5.7	35.0	8.7	26.7	1.5	0.10	27.8	5.5	0.03	30.6	6.6	<0.001	0.001	−3.5	−4.4	−2.1
eStroop AC ratio	executive function	0.83	0.05	0.82	0.08	0.74	0.12	0.87	0.13	0.49	0.83	0.12	0.82	0.80	0.08	<0.001	0.15			
Color Trails 1 (numbers)	attention	29.3	10.6	29.1	8.3	26.4	6.8	25.6	6.7	0.26	24.0	5.9	0.003	22.2	6.4	<0.001	<0.001			
Color Trails 2 (numbers & colors; pink/yellow)	executive function	60.4	15.3	57.3	11.0	58.5	12.6	60.1	12.5	0.95	55.1	10.9	0.31	52.7	11.2	<0.001	0.04	−2.2	−5.8	−3.5
Color Trails 1/2 ratio	executive function	0.49	0.16	0.51	0.15	0.46	0.08	0.43	0.11	0.35	0.44	0.09	0.04	0.43	0.12	<0.001	0.04			
Digit Span Forward	attention	10.3	2.3	11.3	2.0	11.5	1.8	10.9	2.3	0.52	11.4	2.0	0.78	11.0	1.6	<0.0001	0.50			
Digit Span Backward	executive function	6.6	2.1	7.3	2.0	6.8	2.0	6.6	1.3	1.00	7.4	1.9	0.70	7.2	1.7	<0.001	0.36	0.2	0.4	0.6
Digit Span Total	executive function	16.3	3.5	18.4	3.4	18.3	2.6	18.0	3.7	0.17	18.9	3.4	0.39	18.0	2.5	<0.001	0.73			
Digit Span B/F ratio	executive function	0.64	0.11	0.65	0.15	0.60	0.20	0.61	0.10	0.69	0.66	0.15	0.80	0.67	0.18	<0.001	0.19			

## Data Availability

The raw data supporting the conclusions of this article will be made available by the authors on request.

## Abbreviations

The following abbreviations are used in this manuscript:

ADHD	Attention Deficit Hyperactivity Disorder
iPACES	interactive Physical and Cognitive Exercise System
